# MINERVA API and plugins: opening molecular network analysis and visualization to the community

**DOI:** 10.1093/bioinformatics/btz286

**Published:** 2019-04-25

**Authors:** David Hoksza, Piotr Gawron, Marek Ostaszewski, Ewa Smula, Reinhard Schneider

**Affiliations:** 1 Luxembourg Centre for Systems Biomedicine (LCSB), University of Luxembourg, Belvaux L-4367, Luxembourg; 2 Department of Software Engineering, Faculty of Mathematics and Physics, Charles University, Prague 118 00, Czech Republic

## Abstract

**Summary:**

The complexity of molecular networks makes them difficult to navigate and interpret, creating a need for specialized software. MINERVA is a web platform for visualization, exploration and management of molecular networks. Here, we introduce an extension to MINERVA architecture that greatly facilitates the access and use of the stored molecular network data. It allows to incorporate such data in analytical pipelines via a programmatic access interface, and to extend the platform’s visual exploration and analytics functionality via plugin architecture. This is possible for any molecular network hosted by the MINERVA platform encoded in well-recognized systems biology formats. To showcase the possibilities of the plugin architecture, we have developed several plugins extending the MINERVA core functionalities. In the article, we demonstrate the plugins for interactive tree traversal of molecular networks, for enrichment analysis and for mapping and visualization of known disease variants or known adverse drug reactions to molecules in the network.

**Availability and implementation:**

Plugins developed and maintained by the MINERVA team are available under the AGPL v3 license at https://git-r3lab.uni.lu/minerva/plugins/. The MINERVA API and plugin documentation is available at https://minerva-web.lcsb.uni.lu.

## 1 Introduction

One of the goals of systems biology is to model molecular networks of biological systems. Complexity of such networks depends on their size and the type of represented processes and may range from individual reactions, molecular pathways, maps of contextualized mechanisms and pathways (e.g. disease maps; Fujita *et al.*, 2014; Kuperstein *et al.*, 2015), to models of whole systems (e.g. human metabolism; Noronha *et al.*, 2017). With the growing complexity of the networks it becomes increasingly difficult to interpret the encoded processes or cross-link their content of other data sources, e.g. databases or experimental data. Therefore, to efficiently interpret and harvest knowledge stored in complex molecular networks, a support from computational pipelines is needed. An important aspect of such pipelines is visual analytics, as it offers a convenient entry point for combining knowledge represented in diagrams with data.

MINERVA Platform (Gawron *et al.*, 2016) is a tool for web-based visualization and exploration of complex systems biology, e.g. disease maps (Mazein *et al.*, 2018). MINERVA enables visualization of expression data and cross-linking of molecules to external resources, including drug, chemical or microRNA interaction databases. However, such a platform needs to account for a plethora of different analytical and visualization techniques in order to support translational medicine research.

Here we describe two new functionalities of MINERVA addressing this issue: (i) an Application Programming Interface (API) for programmatic access and manipulation of hosted data, and (ii) a plugin architecture allowing to flexibly extend existing functionality of the web interface.

## 2 MINERVA platform

MINERVA was developed to host and interpret complex molecular interaction networks, e.g. disease maps. As such it includes mechanisms to explore and analyze large molecular networks, including semantic zooming, querying mechanisms or the ability to host big molecular network as a set of smaller, interlinked submaps. Another functionality allows to map which entities in the network appear as targets in external resources such as drugs, chemicals or microRNAs. Network elements can be annotated by links to external resources, in particular those supported by identifiers.org (Juty *et al.*, 2012). Finally, users can upload their own experimental datasets and overlay them over the network.

### 2.1 API

MINERVA is as a client–server application with web-based client. Since a recent update (version 12), the entire client–server communication happens via REST API. This gives users a programmatic access to all of the above-introduced functionality, which has been previously accessible only via the web interface. This includes the ability to obtain information about any molecular entity or reaction, including their annotations, to query external resources (e.g. all drugs interacting with a protein), to upload user data (e.g. gene expression or variation) as visual overlays and obtain their intersection with the network. Using the API, users are thus given the possibility to access any molecular data from any network running over MINERVA and even compare data across different networks. With sufficient privileges the API can be used to manage and update hosted networks.

To query the API of any MINERVA installation, one can either use functions provided in the programing language of her choice or simply use a command line tool such as cURL.

### 2.2 Plugin architecture

**Fig. 1.**
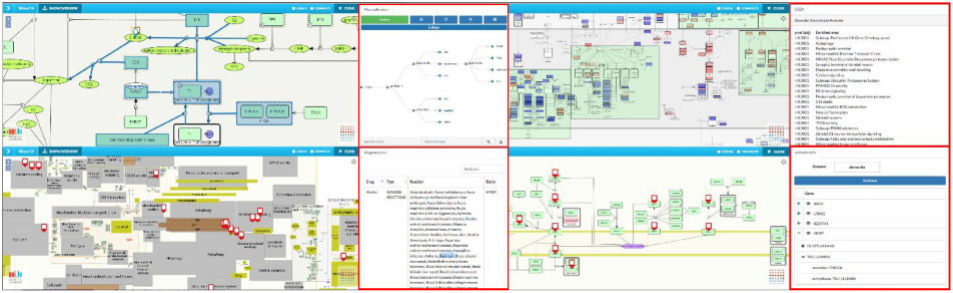
Plugin examples (red boxes) with Parkinson’s disease map (https://pdmap.uni.lu/). Left top: Network traversal plugin with exploration starting in ATG7 gene highlighting traversed parts of the network. Right top: Gene set enrichment plugin showing enriched pathways for differentially expressed genes in Parkinson’s disease substantia nigra post-mortem tissues. Left bottom: Adverse drug reactions plugin showing proteins which are known to be interacting with drugs having respiratory failure as one of their adverse reactions. Right bottom: Variation plugin showing known variants associated with Alzheimer’s disease and overlaying one of them (transferrin) over the iron metabolism submap (Color version of this figure is available at *Bioinformatics* online.)

Starting version 12, MINERVA also implements a plugin architecture which makes existing functionality extendible via plugins. Plugins are JavaScript files which need to implement a specific registration function, which is called on the plugin load and which is passed an object representing a proxy to given MINERVA instance. The plugin appears in a dedicated area of the web interface (see Fig. 1) and this area becomes available via the proxy to the plugin. The main purpose of the proxy is to provide the access to the MINERVA instance. Specifically, via the proxy, the plugins can retrieve data from the map, interact with it and listen to its events. The retrieved data include information about the instance, about entities in the map and their annotations or information about uploaded experimental data (overlays). Map interaction functionality includes (de)highlighting molecules, compartments and pathways, focusing regions of the map or controlling visibility of overlays. Events to which a plugin can listen include search queries, showing and hiding overlays or zoom, resize and panning events. Functionalities not handled by the proxy are still accessible via the API and thus available to the plugins.

A plugin can be loaded into the application by providing a web address from which the plugin can be fetched. MINERVA downloads the plugin code and imports it by running the registration function. The ability to load a plugin is not limited by user access.

The list of plugins developed by the MINERVA team is accessible at https://git-r3lab.uni.lu/minerva/plugins, and currently includes plugins as below.

- Interactive tree traversal of a network: the plugin displays the neighbors of a selected node as a tree, allowing the user to iteratively expand selected nodes, facilitating exploration of complex networks.
- Gene set enrichment analysis: in maps with defined pathway areas, the plugin retrieves active data overlays and performs enrichment analysis, highlighting pathways significantly enriched for these, possibly user-provided, data overlays.
- Mapping adverse drug reactions: this plugin links an external data file to the corresponding map elements. Targets of drugs with identified adverse reactions (Demner-Fushman *et al.*, 2018) are shown in the map, and can be filtered on-the-fly.
- Mapping known disease variants: this plugin links to the Proteins API (Nightingale *et al.*, 2017) and indicates genes with variants associated with a given disease.

Moreover, the repository contains a starter kit—an introduction plugin showcasing the plugin possibilities using simple examples—making the plugin development easier for new developers.

## 3 Summary

New functionalities introduced in MINERVA can help users in two ways. First, the API allows to include the originally purely web-based functionality into high-throughput computational pipelines. Second, whenever a custom visual interpretation is required, users can benefit from the existing web interface and easily extend its capabilities via the plugins architecture. The plugin examples indicated above demonstrate the potential for different analytical pipelines available in MINERVA. We believe that the combination of API and plugins architecture makes MINERVA extremely versatile systems biology tool empowering researchers to employ large molecular network analysis in a wide range of translational medicine and drug-discovery use cases. The documentation of the new features can be accessed at https://minerva.pages.uni.lu/doc/api/ and the list of available plugins at https://git-r3lab.uni.lu/minerva/plugins/.

*Conflict of Interest*: none declared.
